# Activation of the Nucleus Taeniae of the Amygdala by Umami Taste in Domestic Chicks (*Gallus gallus*)

**DOI:** 10.3389/fphys.2022.897931

**Published:** 2022-05-26

**Authors:** Francesca Protti-Sánchez, Carlos Daniel Corrales Parada, Uwe Mayer, Hannah M. Rowland

**Affiliations:** ^1^ Max Planck Institute for Chemical Ecology, Jena, Germany; ^2^ Institute for Biology, Karl-Franzens-University Graz, Graz, Austria; ^3^ Center for Mind/Brain Sciences (CIMeC), University of Trento, Rovereto, Italy

**Keywords:** reward, bitter, taste perception, Immediate early genes (IEGs), accumbens (NAc), lateral septum (LS), c-Fos

## Abstract

In chickens, the sense of taste plays an important role in detecting nutrients and choosing feed. The molecular mechanisms underlying the taste-sensing system of chickens are well studied, but the neural mechanisms underlying taste reactivity have received less attention. Here we report the short-term taste behaviour of chickens towards umami and bitter (quinine) taste solutions and the associated neural activity in the nucleus taeniae of the amygdala, nucleus accumbens and lateral septum. We found that chickens had more contact with and drank greater volumes of umami than bitter or a water control, and that chicks displayed increased head shaking in response to bitter compared to the other tastes. We found that there was a higher neural activity, measured as c-Fos activation, in response to umami taste in the right hemisphere of the nucleus taeniae of the amygdala. In the left hemisphere, there was a higher c-Fos activation of the nucleus taeniae of the amygdala in response to bitter than in the right hemisphere. Our findings provide clear evidence that chickens respond differently to umami and bitter tastes, that there is a lateralised response to tastes at the neural level, and reveals a new function of the avian nucleus taeniae of the amygdala as a region processing reward information.

## Introduction

Taste perception provides animals with information about the quality of food via five basic tastes: sweet, umami, bitter, salty and sour (Jiang et al., 2012). Bitter can indicate toxic and harmful substances, and aversion to bitter tastes likely evolved as a protective mechanism (Davis et al., 2010). Umami, on the other hand, reflects amino acids in protein (Chandrashekar et al., 2006), and is known to increase appetite, but also to increase satiety (Masic and Yeomans, 2014). Research on the mechanism(s) involved in taste perception will increase our understanding of how animals use taste to make adaptive foraging decisions.

To fully understand taste perception requires comparative physiological studies ranging from receptor mechanisms to brain circuitry, but also psychophysical studies that quantitatively describe the perceptual output of the system (Green, 2021). These types of research can lead to the discovery of new phenomena such as the functional loss or neofunctionalization of taste receptors (Baldwin et al., 2014), which generate new insights and hypotheses about the mechanisms and evolution of taste perception (Zhao et al., 2010; Jiang et al., 2012; Toda et al., 2021). The comparative genetic linkage between taste perception and feeding specializations are well studied (Li and Zhang, 2014; Wang and Zhao, 2015; Liu et al., 2016), whereas the cognitive mechanisms that underlie taste perception in animals other than humans, non-human primates, and rodents is less studied.

Among birds, domestic chickens (*Gallus gallus domesticus*) are probably the most intensively investigated species with regards to taste receptor physiology and associated behaviour, which can be attributed to their commercial importance (for a review see Roura et al., 2013). Chickens lack the receptor for sweet taste (Cheled-Shoval et al., 2015), but possess umami and bitter receptors in their oral cavity (Shi and Zhang, 2006; Yoshida et al., 2015). The behavioural responses of chickens to sour, salty, umami, and bitter can be observed during the first post hatch day (Ganchrow et al., 1990). The typical response to bitter includes prolonged head shaking, beak wiping and sticking out the tongue (Nicol, 2004). Chicks also develop conditioned taste aversions and exhibit foraging biases in response to bitter (Skelhorn and Rowe, 2005a; Skelhorn and Rowe, 2005b; Skelhorn et al., 2008). Umami, on the other hand, is palatable for chickens (Roura et al., 2013; Liu et al., 2018; Niknafs and Roura, 2018) and it is suggested that chicks may perceive umami as a salty-and sweet-like taste (Yoshida et al., 2018).

While there has been some research into the neural mechanisms underlying reward in domestic chickens (Yanagihara et al., 2001; Izawa et al., 2002; Aoki et al., 2003; Matsushima et al., 2003; Ichikawa et al., 2004; Amita and Matsushima, 2014), there has been less attention given to the brain regions involved in palatable taste perception. In mammals, the pathway for the gustatory sensory information runs through the nucleus of the solitary tract to the parabranchial nucleus, which in turn projects to various forebrain regions, including the amygdaloid arcopallium and nucleus accumbens (Arends et al., 1988; Wild et al., 1990; Berk et al., 1993). Palatable taste perception involves the mesolimbic rewards system (Dayan and Balleine, 2002; Ramírez-Lugo et al., 2007; Berridge et al., 2009), which includes the nucleus accumbens, ventral pallidum, basolateral and medial amygdala (Ramírez-Lugo et al., 2006; Ramírez-Lugo et al., 2007; Wassum et al., 2009; Berridge and Kringelbach, 2013; Baumgartner et al., 2021; Hu et al., 2021). Although the mesolimbic reward system is highly conserved in vertebrates (O’Connell and Hofmann, 2011), investigating whether these brain areas are also involved in taste perception in domestic chicks will add to our understanding of comparative taste perception and reward behaviour.

**FIGURE 1:**
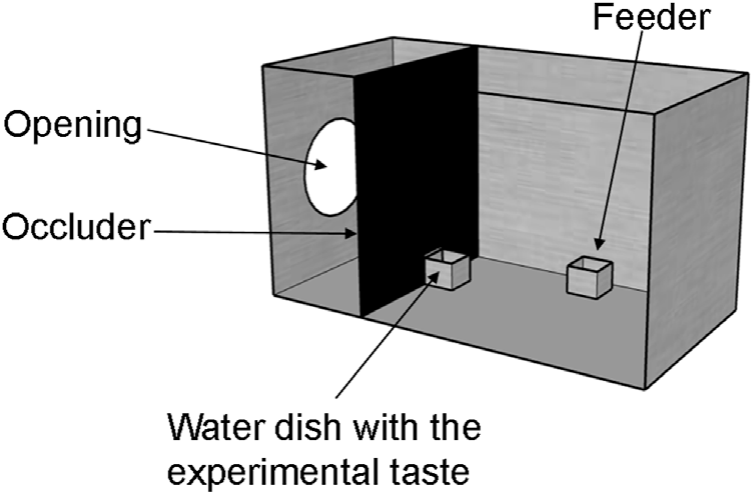
Experimental setup. Chicks were habituated in metal cages with food and water *ad libitum*. Prior to the presentation of the experimental taste, chicks were water deprived for 45 min by removing their water dish through an opening in the occluder. Then, an identical water dish with the experimental taste was presented for 10 minutes. A camera was placed above each cage to record chicks behavioural responses to the taste.

**FIGURE 2:**
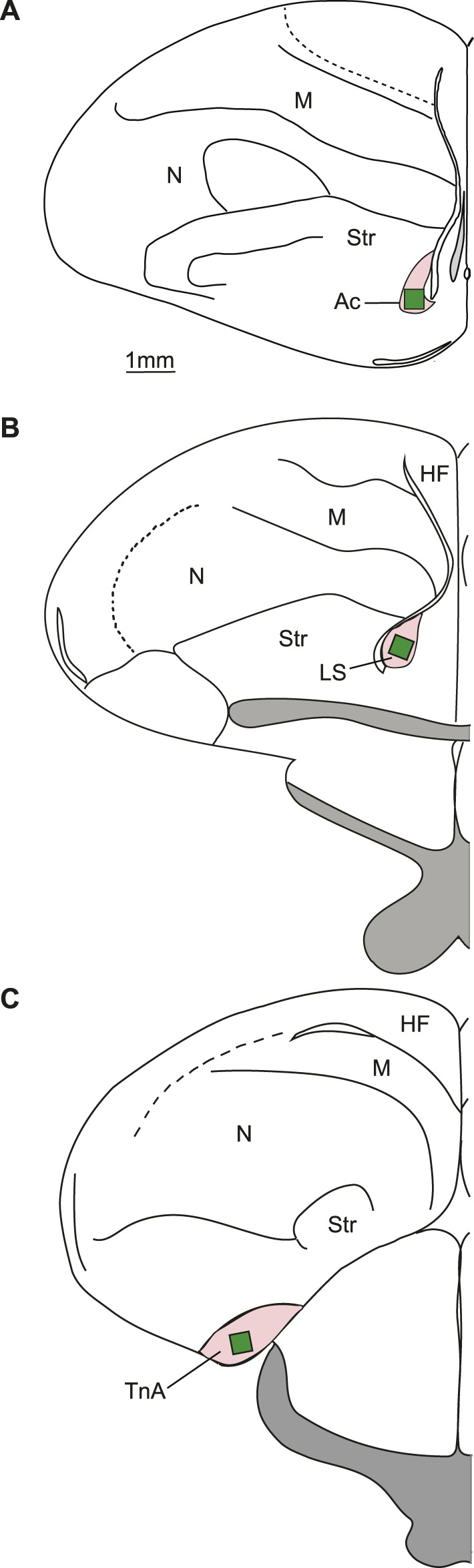
Typical placements of cell counting zones (green rectangles) in the regions of interest (pink). **(A)** Schematic representation of a coronal section at the level of the nucleus accumbens. **(B)** Coronal section at the level of the lateral septum. **(C)** Coronal section at the level of nucleus taeniae of the amygdala. TnA—nucleus taeniae of the amygdala; LS—lateral septum; Ac—nucleus accumbens; HF—hippocampal formation; M—mesopallium; N—nidopallium; Str - Striatum.

Here we present three different groups of young domestic chicks with either a solution containing monosodium glutamate and inosine 5′-monophosphate (umami taste), quinine solution (bitter taste), or to pure water (as a control) (Figure 1). Behavioural reactions to these stimuli were measured in terms of: 1) frequency of contact with the solution, 2) volume of liquid consumed, 3) frequency of head shakes and 4) beak cleaning episodes. Brain activation was measured by immunohistochemical detection of the Immediate Early Gene (IEG) product c-Fos in three interconnected subpallial brain regions: nucleus taeniae of the amygdala (TnA), nucleus accumbens (Ac) and lateral septum (LS) (Figure 2) that may directly modulate taste-responsive neurons of the parabrachial complex through an anatomical pathway using neurotensin (Bálint et al., 2011; Bálint et al., 2016), or receive gustatory information indirectly.

## Results

### Behaviour

**FIGURE 3:**
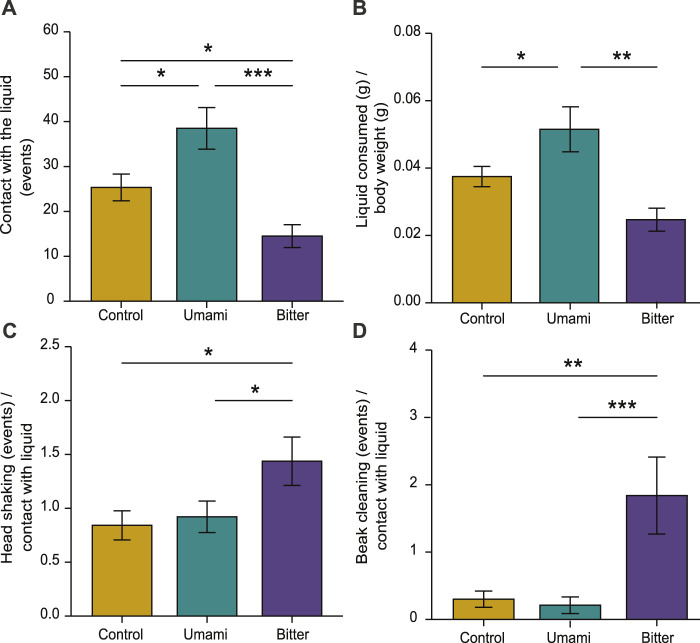
Behavioural responses of chicks according to experimental tastes. **(A)** Contact with the liquid, **(B)** liquid consumed/body weight, **(C)** head shaking in relation to contact with the liquid and **(D)** beak cleaning in relation to contact with the liquid. Bar plots indicate mean ± s.e.m. (∗*p* < 0.05, ∗∗*p* < 0.01, ∗∗∗*p* < 0.001).

Contact with the liquid significantly varied among treatments (ANOVA: F_33,2_ = 11.76, *p* < 0.001). Chicks had more contact with umami (mean ± s.e.m.: 38.5 ± 4.63) than with bitter (14.5 ± 2.54; *p* < 0.001) or control (25.3 ± 2.98; *p* = 0.012), and more contact with control than with bitter (*p* = 0.03) (Figure 3A). The amount of liquid consumed was also significantly different among treatments (F_33,2_ = 8.25, *p* = 0.001). Chicks consumed significantly more umami (0.05 ± 0.003) than control (0.03 ± 0.002; *p* = 0.041) or bitter (0.02 ± 0.003; *p* < 0.005) (Figure 3B). Head shaking was significantly different among treatments (F_33,2_ = 3.47, *p* = 0.042). Chicks shook their head significantly more frequently after having contact with bitter (1.43 ± 0.22) than with umami (0.92 ± 0.14; *p* = 0.04) or control (0.84 ± 0.13, *p* = 0.02) (Figure 3C). Beak cleaning was also significantly different among treatments (Kruskal–Wallis: X^2^ = 11.629, d.f. = 2, *p* = 0.002). Chicks cleaned their beak more frequently after having contact with bitter (1.83 ± 0.57) than with control (0.30 ± 0.12; *p* = 0.008) or umami (0.21 ± 0.12; *p* = 0.001) (Figure 3D).

### Brain Activity

**FIGURE 4:**
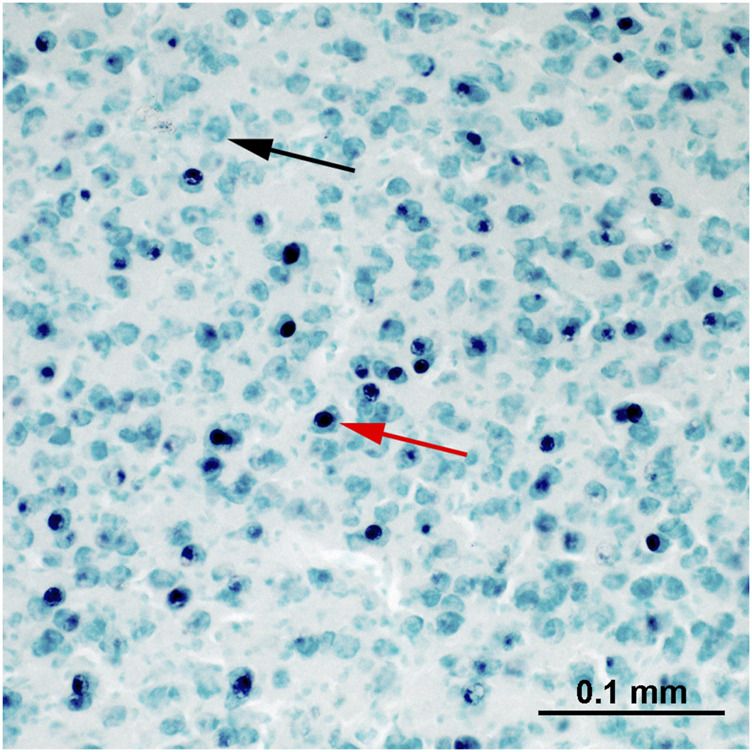
An example of c-Fos staining within the nucleus taeniae of the amygdala. The nuclei of c-Fos immunoreactive cells are stained black (red arrow). The c-Fos-negative cells (black arrow) are green due to the methyl-green counterstaining.

All 36 brains (*n* = 12 in each treatment) were successfully stained for c-Fos (Figure 4). However, the accumbens region of one brain from the quinine group was damaged and was excluded from further analysis.

**FIGURE 5:**
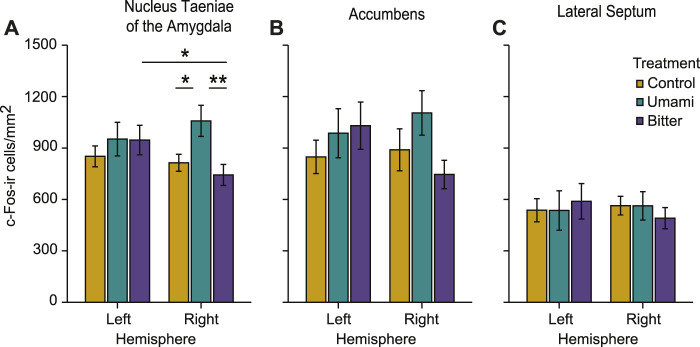
Measured c-Fos-ir (immunoreactive) cell densities of the different treatments across regions of interest, in the left and right hemisphere. **(A)** nucleus taeniae of the amygdala, **(B)** nucleus accumbens, and **(C)** lateral septum. Bar plots indicate mean ± s.e.m. (∗*p* < 0.05, ∗∗*p* < 0.01).

In nucleus taeniae of the amygdala there was a significant interaction between treatment and hemisphere (*p* = 0.008), meaning that in one of the hemispheres, treatment had an effect on c-Fos activation as revealed by ANOVA (Table 1). Only in the right nucleus taeniae was c-Fos activation higher in the umami group (Mean ± s.e.m: 1058.33 ± 90.92 cells/mm^2^) than in the control (813.75 ± 49.38 cells/mm^2^; *p* = 0.032), or bitter (742.81 ± 61.39 cells/mm^2^; *p* = 0.007) (Table 1). Such differences between treatments were not present in the left nucleus taeniae (control 851.45 ± 60.73 cells/mm^2^; umami: 952.08 ± 97.90 cells/mm^2^; bitter: 946.35 ± 86.28 cells/mm^2^) (Figure 5A). Moreover, an additional lateralised response was found in nucleus taeniae. c-Fos activation of the bitter group was higher in the left nucleus taeniae than in the right (*p* = 0.014). Control and umami treatments did not present such differences (Table 1).

**TABLE 1:** Summary of the statistical results of brain activation.

Brain region	Treatment	Hemisphere	Interaction
Nucleus taeniae	F_(1,33)_=1.699, *p =* 0.199	F_(1,33)_=1.266, *p =* 0.269	F_(2,33)_=5.620, *p =* 0.008*
Lateral septum	F_(2,33)_=0.66, *p =* 0.994	F_(1,33)_=0.950, *p =* 0.759	F_(2,33)_=0.749, *p =* 0.481
Accumbens	F_(2,32)_=0.766, *p =* 0.473	F_(1,32)_=0.215, *p =* 0.646	F_(2,32)_=3.075, *p =* 0.060
* indicates the significant interaction based on which the post hoc analyses reported below were performed.			
Treatments	Left hemisphere	Right hemisphere	Left vs. Right
Bitter vs. Umami	t_(22)_=-0.21, *p =* 0.984	t_(22)_=-2,955, ***p =* 0.007**	Control: t_(11)_=0.540, *p =* 0.600
Bitter vs. Control	t_(22)_=0.812, *p =* 0.426	t_(22)_=-1.006, *p =* 0.325	Umami: t_(11)_=-1.887, *p =* 0.086
Umami vs. Control	t_(22)_=0.699, *p =* 0.492	t_(22)_=2.292, ***p =* 0.032**	Bitter: t_(11)_=2.905, ***p =* 0.014**

Significant effects are highlighted in bold (*p* ≤ 0.05).

In the other two brain regions (accumbens and lateral septum), there were no main effects of treatment or hemisphere and no interactions (Table1; Figure 5). However, patterns of activation qualitatively similar to the right nucleus taeniae of the amygdala were present also in right accumbens (control: 889.89 ± 122.50 cells/mm^2^; umami: 1104.99 ± 129.71 cells/mm^2^; bitter: 745.59 ± 82.49 cells/mm^2^), although the interaction was not significant at the alpha 0.05 level (*p* = 0.06, Table 1). The activation pattern in the left accumbens did not differ (control 848.30 ± 97.34 cells/mm^2^; umami: 986.27 ± 143.08 cells/mm^2^; bitter: 1030.36 ± 137.95 cells/mm^2^), nor did the left lateral septum (control: 536.97 ± 67.73 cells/mm^2^; umami: 535.52 ± 115.02 cells/mm^2^; bitter: 589.06 ± 103.74 cells/mm^2^), or right lateral septum (control: 563.67 ± 54.40 cells/mm^2^; umami: 562.57 ± 82.53 cells/mm^2^; bitter: 490.52 ± 62.03 cells/mm^2^).

### Correlations

There were no significant correlations between c-Fos activation and any of the measured behaviours in any of the brain regions analysed (Table S1).

## Discussion

We show that the avian nucleus taeniae of the amygdala responds to gustatory information: at the brain level, c-Fos activation was higher in the right nucleus taeniae in chicks that consumed umami, compared to those that consumed bitter or water control. This is the first evidence to show that the avian nucleus taeniae of the amygdala responds to umami taste. We also show a clear behavioural preference for umami and an aversion to bitter: chicks consumed more umami than bitter and water, and shook their heads and cleaned their beaks more after contact with bitter than with umami or water (see also Gentle, 1975; Ganchrow et al., 1990; Yoshida et al., 2015; Yoshida et al., 2018). The higher activation in the right nucleus taeniae of the amygdala in response to umami, and the greater consumption of umami is likely indicative of reward processing. The avian nucleus taeniae of the amygdala resembles a homolog brain region to the mammalian medial amygdala (Reiner et al., 2004; Yamamoto et al., 2005; Abellán et al., 2009), which in humans and other non-human primates is associated with reward experiences, such as a palatable tastes (Azuma et al., 1984; De Araujo et al., 2003; Wu et al., 2021). Our results suggest a conserved function of avian nucleus taeniae of the amygdala and the medial amygdala in mammals, and the involvement of the mesolimbic reward network in processing taste perception.

In mice, the medial amygdala is associated with processing reward information, at least in a social context (Hu et al., 2021). And in domestic chickens the nucleus taeniae of the amygdala has been reported to respond to social stimuli (Mayer et al., 2019), to novel environments (Morandi-Raikova and Mayer, 2020) and in phobic reactions to novel foods and objects (Perez et al., 2020). In our study we can exclude neophobia from explaining the response of nucleus taeniae of the amygdala because both bitter and umami tastes were novel, only umami was consumed significantly more by chicks, and only umami induced high activation of the right nucleus taeniae of the amygdala. The reward function of nucleus taeniae of the amygdala is further supported by a similar (although not significant) pattern of activity observed in the accumbens (Figure 5B). Accumbens in vertebrates is involved in processing reward information and plays an important role in learning and motivation (Day and Carelli, 2007). In domestic chicks the role of accumbens in processing reward information has been investigated only in a few studies (e.g. Yanagihara et al., 2001; Izawa et al., 2002; Aoki et al., 2003; Izawa et al., 2003; Ichikawa et al., 2004; Amita and Matsushima, 2014) which have suggested a role in the control of impulsivity when choosing a predictable food reward (Cardinal et al., 2001). Further studies should assess whether avian nucleus taeniae and nucleus accumbens work together in processing food reward information.

We also found higher c-Fos expression in the left nucleus taeniae of the amygdala than in the right in the chicks presented with bitter taste. This could indicate that some lateralised processing of bitter taste is happening in the nucleus taeniae of the amygdala. In humans, the amygdala plays a role in the recognition, evaluation and response to aversive stimuli (Zald et al., 1998). However, an alternative explanation is that this lateralisation pattern in the nucleus taeniae of the amygdala is not related to the recognition of bitter taste at all. Task independent higher expression of c-Fos in the left nucleus taeniae of the amygdala compared to right was present in our previous study (Corrales Parada et al., 2021), and spontaneous lateralisation of c-Fos activity in young chicks without performing any task has also been found in other brain areas (Lorenzi et al., 2019). Therefore, our results could be indicative of a baseline lateralisation of c-Fos expression independent of bitter taste. These explanations could be teased apart by testing chicks with different concentrations of bitter tastes, and different bitter tastants, and comparing the responses to a water control group.

In chicks, accumbens has been proposed to be involved in the formation of aversive taste memories in a passive avoidance learning paradigm (Patterson and Rose, 1992; Stewart and Rusakov, 1995; Csillag, 1999; Rose, 2000; Bálint et al., 2011). In our study, we did not find a response of accumbens to bitter taste. The paradigm used in our study had some important differences to the classic passive avoidance learning in which chicks are naïve to any visual stimuli, and spontaneously peck on a coloured bead and learn to associate the bitter taste with the colour of the bead. In our study, all chicks were habituated with water, and likely formed a positive association of water taste with the water container. Chicks in our study also had restricted access to water prior to the test to motivate them to drink. When we presented the experimental taste in the same container that was used during habituation, it is possible that the chicks anticipated a rewarding outcome. In humans, altering expectancy reduces neural responses of the gustatory cortex to aversive taste when participants were led to believe that a highly aversive bitter taste would be less distasteful than it actually was (Nitschke et al., 2006). If chicks expected a rewarded experience, this could explain why we did not observe activation of the brain regions associated with aversive taste as we expected. The lack of neural response to quinine in our study is in line with what has been reported in rats (Yasoshima et al., 2006), where the rats accumbens showed changes in c-Fos expression only during taste aversion retrieval but not after consumption of quinine hydrochloride. Future studies could compare naïve to experienced chicks in the response of the accumbens to bitter tastes, or present different concentration of quinine to naïve newly hatched chicks and investigate c-Fos activation in earlier stations of the gustatory system, such as nucleus of the solitary tract and parabrachial nucleus. In our study, the lateral septum did not show differences between treatments, which confirms a region-specific activation to rewarding taste in nucleus taeniae. To the best of our knowledge, this area has never been reported to process gustatory information. We also found no correlation between the behaviours expressed and c-Fos activation in any of the measured brain areas, which support the conclusion that exposure to appetitive and aversive taste, is associated with activation of the mesolimbic reward system in birds, and that this represents taste perception and not differences in motoric behaviour.

So far, we have only considered the role of gustatory information as a source of our effects. However, olfactory and gustatory information are usually combined in the sensory experience associated with food consumption. Since birds can perceive olfactory information (Jones and Roper, 1997; Krause et al., 2018), it is important to consider also the potential role of olfaction in our results. Chicks respond to odours predominantly when the right nostril is used (Vallortigara and Andrew, 1994; Rogers et al., 1998; Burne and Rogers, 2002). This has been interpreted as reflecting a predominant use of the right side of the brain to process olfactory information (Vallortigara and Andrew, 1994; Rogers et al., 1998; Burne and Rogers, 2002). The primary olfactory sensory area (the olfactory bulb) is a bilateral structure located within the frontal telencephalon of both hemispheres. The bulb projects to further telencephalic areas, without passing the thalamus (a unique feature of the olfactory pathway). It is reasonable to assume that the right bulb would predominantly project to the right hemisphere. However, projections to contralateral areas have also been reported (Reiner and Karten, 1985; Atoji and Wild, 2014). Intriguingly, the nucleus taeniae of the amygdala receives a direct projection from the olfactory bulb (Reiner and Karten, 1985). Thus, the similar rightward bias found in behavioural olfaction studies and in nucleus taeniae’s responses to umami, could indeed suggest a role of olfactory cues in our results. While we cannot exclude this interpretation, at present there is no evidence that chicks are actually able to smell umami. It is also not known if the nucleus taeniae process odour information at all. To date, only one study investigated if this structure would respond to odours in any bird species (Golüke et al., 2019). While in this study hippocampus responded to social odours, no evidence was found for the involvement of nucleus taeniae in odour processing (Golüke et al., 2019). Moreover, it has been shown that in another galliform species, gustatory information plays a more prominent role than olfactory information in responses to aposematic signals associated with aversive taste (Marples et al., 1994). Thus, while we cannot exclude an involvement of olfactory information in the activation of nucleus taeniae, we still believe that gustatory information played a major role in this effect. Future studies should be devoted to clarify this issue, e.g., by testing anosmic animals. In any case, whether through purely gustatory information or a combination of taste and olfaction, our results demonstrate that exposure to umami was a rewarding experience for chicks and that nucleus taeniae is involved in the processing of this rewarding information.

In conclusion, we show that umami taste is associated with higher activation of the right nucleus taeniae of the amygdala in domestic chicks. Our results allows us to speculate that reward processing in birds nucleus taeniae is an ancestral function shared with the mammalian medial amygdala and provides new insights into the regulation of gustatory rewards.

## Methods

### Subjects

We tested 36 four days old female chicks (*Gallus gallus domesticus*) of the Aviagen ROSS 308 strain. Fertilized eggs were obtained from a commercial hatchery (CRESCENTI Societ`a Agricola S.r.l. –Allevamento Trepola–cod. Allevamento127BS105/2). Eggs were incubated from embryonic day one (E0) to seventeen (E17) in complete darkness. On the morning of E18 to the evening of E19, eggs were light stimulated following (Lorenzi et al., 2019). This procedure has an important impact on the lateralisation of chicks visual system (Rogers, 1982; Rogers and Sink, 1988; Rogers, 1990; Rogers and Bolden, 1991; Rogers and Deng, 1999), on the maturation of visual responses (Costalunga et al., 2021) and on several aspects of chicks behaviour (Daisley et al., 2009; Salva et al., 2009). Chicks hatched on E21 in darkness at a constant temperature of 37.7°C and a humidity of 60%. On the day of hatching, chicks were placed in individual metal cages (28 cm× 32 cm× 40 cm; W × H × L), where they were housed for three days with food and water *ad libitum* at a room temperature of 30–32°C and light conditions of 14 h light and 10 h dark.

### Apparatus

On the third day after hatching (24 h prior to the experiment), chicks were taken to the experimental room and housed individually in metal cages (Figure 1), identical to their home cages. The experimental cages differed to the home cage only by the addition of a black plastic wall (occluder) that allowed us to make two compartments in the cage. Chicks were located on the larger section of the cage (25 cm L); while the smaller section (15 cm L) was used for keeping the water dishes separated from the chicks until the start of the experiment, and prevented chicks from observing the experimenter (see below). The two compartments were connected by a closable opening (5 × 4.8 cm) in the plastic wall. Above every cage (76 cm), there was a video camera (Microsoft LifeCam Cinema for Business) for recording the experiment. Illumination of the cages was provided by top lights (25 W warm light) at 67.5 cm above the cage, while the rest of the experimental room was dark.

### Habituation

Chicks were left in their individual experimental cages for 24 h prior to the experiment with water and food *ad libitum*. Room temperature was 26.4–28.7°C, humidity 24–41%, and light conditions of 14 h light and10 h dark.

### Experiment

For the experiment, we assigned 12 chicks to each treatment. Treatments consisted of three experimental tastes (control, umami and bitter). We used water as a control, since chicks were habituated to drink it. As a palatable taste, we used umami in a combination of 2.5% monosodium glutamate (MSG, AJI-NO-MOTO >99%, CEE: E621, France) + 0.25% inosine 5′-monophosphate (IMP, ≥98%, Sigma-Aldrich I2879-1G diluted in water). Yoshida et al. (2018) showed that at this concentration, MSG is an enhancer of the umami taste (IMP) in chicks. As an unpalatable bitter taste, we used quinine (Alfa Aesar A10459 99%) 10 mM dissolved in water. The experimental solutions were prepared the day prior to the experiment and kept in the experimental room to adjust their temperature to the room temperature. Experimental tastes were colourless and were presented to chicks in identical containers with 80 ml solution in each. Before the experiment chicks were deprived of water for 45 min. After this period, the experimental taste was presented for 10 min, the behaviour of the chicks was recorded, and after that, the experimental container was removed. The containers with the experimental solutions were weighted before and after the experiment in order to estimate the amount of liquid consumed by each chick. We ran the experiment in 4 days, testing nine chicks per day. Every testing day, we assigned three individuals to each treatment in randomized order.

### Video Analysis

We analysed the videos for seven minutes after the first contact with the experimental taste. In this period, we recorded the total number of the following events: contact with the liquid (when chicks peck the experimental solution), head shaking, and beak cleaning. The category ‘beak cleaning’ includes two behaviours: beak wiping, when the animal wipes its beak on the floor and beak scratching, performed with a foot. Video analysis was conducted blinded to the experimental conditions using the VLC media player software (v. 3.0.12).

### Histology and Immunohistochemistry

Seventy min after the first contact with the experimental taste, all chicks were weighted and received a lethal dose (0.8 ml) of a ketamine/xylazine solution (1:1 ketamine 10 mg/ml + xylazine 2 mg/ml). They were then perfused transcardially with phosphate-buffered saline (PBS; 0.1 mol, pH = 7.4, 0.9% sodium chloride, 4°C) and paraformaldehyde (4% PFA in PBS, 4°C). The head was post-fixed and stored in 4% PFA for at least 8 days until processing. All the following brain processing steps were performed blind to the experimental treatments. To ensure that the subsequent coronal brain sections would have the same orientation (°45), brains were removed from the skulls following the procedures described in the chick brain atlas (Kuenzel and Masson, 1988). The left and the right hemispheres were separated and processed independently. Each hemisphere was embedded in a 7% gelatine in PBS solution containing egg yolk at 40°C and post-fixated for 48h at 5°C in 20% sucrose in 4% PFA/PBS and further 48 h in 30% sucrose in 0.4% PFA/PBS solution. Four series of 45 μm coronal sections were cut on a Cryostat (Leica CM1850 UV) at −20°C. Two out of the four series were collected in cold PBS. The sections of the first series were used for labelling. The sections of the second series were kept as backup. For free-floating immunostaining, sections were incubated in 0.3% H2O2 in PBS for 20 min to deplete endogenous peroxidase activity. Between every of the following steps of the procedure the sections were washed in PBS. After the sections were treated with 3% normal goat serum (S-1000, Vector Laboratories, Burlingame, CA) in PBS for 30 min at room temperature, they were transferred to the c-Fos antibody solution (c-Fos antibody, 1:2000; rabbit, polyclonal K-25, Santa Cruz Biotechnology, Santa Cruz, CA) containing 0.1% Bovine Serum Albumin (BSA, SP-5050, Vector Laboratories) in PBS and incubated for 48 h at 5°C on a rotator. The secondary antibody reaction was carried out using a biotinylated anti-rabbit solution (1:200, BA- 1000, Vector Laboratories) in PBS for 60 min at room temperature on a rotator. The ABC method was used for signal amplification (Vectastain Elite ABC Kit, PK-6100, Vector Laboratories). Cells with concentrated c-Fos protein were visualized with the VIP substrate kit for peroxidase (SK-4600, Vector Laboratories), at a constant temperature of 25°C for 25 min. This produced a dark purple reaction product confined to the nuclei of c-Fos immunoreactive (-ir) cells. Brain sections of right and left hemispheres were mounted with the same orientation on gelatine-coated slides, enabling the coder to be blind to the hemisphere’s identity while counting. Slides were then dried at 50°C, counterstained with methyl green (H-3402, Vector Laboratories) and cover slipped with Eukitt (FLUKA).

### Brain Anatomy

Brain sections were analysed blind to the experimental condition and hemispheres. c-Fos-ir cells were stained purple-black and were easily discerned from the background and non-activated cells, which were stained light-green (Figure 4). Photos were taken with a Zeiss microscope (objective magnification: ×20 with a numerical aperture of 0.5) connected to a digital camera (Zeiss AxioCam MRc5) and the Zeiss imaging software ZEN. Exposure time of the camera and the light conditions of the microscope were kept constant for all photos, while the contrast was slightly adjusted for individual photos to match their visual appearance if it was needed.

To analyse brain activation within the regions of interest a spot with the highest number of c-Fos-ir cells was chosen, by visual observation under the microscope. Then a counting area (400 × 400 µm) was positioned over this spot by keeping a minimum distance of 10 µm from the borders. A snapshot was taken, cropped around the counting area and saved. Automatic counting of the c-Fos immunoreactive cells was performed using the “analyse particle” function of the ImageJ software (Schneider et al., 2012). All photos were analysed with a predefined macro, where the image was transformed into 8bit, threshold was set to 120, circularity of particles to 0.5–1.0 and particle size to 2–200.

For the analysis of nucleus taeniae of the amygdala five sections of both hemispheres were selected from an area corresponding to the A8.8 to A6.4 of the brain atlas (Kuenzel and Masson, 1988) (Figure 2C). To quantify c-Fos-ir cells in accumbens (Bálint et al., 2016), five sections of both hemispheres were selected from A10.0 to A9.2 (Kuenzel and Masson, 1988; Figure 2A). Lateral septum was measured on five selected sections from A8.8 to A7.6 (Kuenzel and Masson, 1988) in the lower half of septum starting from the ventral border of the lateral ventricle (Figure 2B). The lateral septum was further delineated from the medial septum based on anatomical landmarks that are visible after methyl green counterstain. The cell densities obtained from the different brain sections were averaged to estimate overall activity in each measured area. These individual bird means were employed for further statistical analysis.

### Statistical Analysis

All statistical analysis were conducted with R v. 4.0.4 (R Core Team, 2021). We analysed whether behavioural responses varied according to treatment. The response variables were contact with the liquid, liquid consumed per body weight, head shaking and beak cleaning. The raw values were divided by the number of contact with liquid episodes. This allowed to reveal how much the contact with liquid elicited the behaviours of interest. ANOVA was run on the first three variables with Fischer’s LSD tests for *post-hoc* analysis. Given that for beak cleaning the residuals were not normally distributed (Shapiro-Wilk test, W = 0.7382, *p* < 0.05), even after square root or logarithmic transformation, a non-parametric Kruskal–Wallis test was used. Significance between the comparisons was assessed with *post-hoc* Dunn tests.

In order to test whether c-Fos activation (response variable) varied according to treatment and hemisphere (factors), we conducted a repeated measures ANOVA for each brain region. We used a between-subject factors with three levels (Treatment: control, umami, bitter), and within-subject factor with two levels (Hemisphere: left and right). Given that for nucleus taeniae of the amygdala and accumbens residuals were not normally distributed (Shapiro-Wilk Test; nucleus taeniae: W_71_ = 0.955, *p* = 0.013; accumbens: W_71_ = 0.952, *p* = 0.009), we used a square root transformation. This procedure significantly improved the normality of residuals (nucleus taeniae: W_71_ = 0.979, *p* = 0.272; accumbens: W_71_ = 0.982, *p* = 0.393), and further analysis were conducted with transformed data. Between treatment comparisons were assessed with independent *post-hoc* t-tests, while between hemispheres comparisons for each treatment was assessed with paired t-tests. In order to test whether behaviour and motoric activity is correlated with c-Fos expression, we performed Pearson correlations between c-Fos expression in the brain regions analysed and the behaviours recorded for each of the treatments and hemispheres. All figures were created or postprocessed using Adobe Photoshop and Illustrator software.

## Data Availability

The original contributions presented in the study are included in the article/Supplementary Material, further inquiries can be directed to the corresponding author.
