# Supplementary material for: Activation of the Nucleus Taeniae of the Amygdala by Umami Taste in Domestic Chicks (Gallus gallus)
Source: Front Physiol. 2022 May 26;13:897931. doi: 10.3389/fphys.2022.897931 (PMC9178096; doi:10.3389/fphys.2022.897931)
Supplement: Supplementary file 1 [file Table1.docx]

**Table S1:** Pearson correlations between behaviour and c-Fos activation in the different brain regions

| Brain region | Hemi-sphere | Groups | Contact with liquid | Liquid consumed | Head shaking | Beak cleaning |
| --- | --- | --- | --- | --- | --- | --- |
| TnA | Left | Control | R=-0.41, p=0.18 | R=-0.07, p=0.83 | R=-0.16, p=0.62 | R=0.24, p=0.46 |
|  |  | Umami | R=-0.13, p=0.68 | R=0.19, p=0.56 | R=-0.45, p=0.14 | R=-0.36, p=0.25 |
|  |  | Bitter | R=0.02, p=0.96 | R=0.03, p=0.94 | R=-0.23, p=0.48 | R=0.02, p=0.96 |
|  | Right | Control | R=-0.05, p=0.88 | R=0.08, p=0.8 | R=-0.27, p=0.39 | R=0.012, p=0.97 |
|  |  | Umami | R=0.05, p=0.88 | R=0.02, p=0.95 | R=-0.17, p=0.59 | R=-0.39, p=0.22 |
|  |  | Bitter | R=-0.26, p=0.41 | R=-0.23, p=0.47 | R=-0.13, p=0.7 | R=-0.15, p=0.64 |
| Ac | Left | Control | R=-0.09, p=0.77 | R=-0.05, p=0.87 | R=-0.28, p=0.38 | R=0.048, p=0.88 |
|  |  | Umami | R=-0.19, p=0.56 | R=0.39, p=0.21 | R=-0.5, p=0.10 | R=-0.11, p=0.74 |
|  |  | Bitter | R=-0.35, p=0.29 | R=-0.20, p=0.38 | R=-0.27, p=0.43 | R=0.049, p=0.89 |
|  | Right | Control | R=0.38, p=0.22 | R=0.18, p=0.58 | R=0.38, p=0.22 | R=0.063, p=0.85 |
|  |  | Umami | R=-0.13, p=0.69 | R=0.07, p=0.82 | R=-0.34, p=0.28 | R=-0.053, p=0.87 |
|  |  | Bitter | R=-0.23, p=0.47 | R=-0.01, p=0.97 | R=0.006, p=0.99 | R=0.044, p=0.89 |
| LS | Left | Control | R=-0.32, p=0.31 | R=-0.17, p=0.59 | R=0.03, p=0.93 | R=0.26, p=0.42 |
|  |  | Umami | R=-0.15, p=0.63 | R=0.32, p=0.32 | R=-0.53, p=0.07 | R=-0.15, p=0.65 |
|  |  | Bitter | R=-0.26, p=0.41 | R=-0.23, p=0.48 | R=-0.28, p=0.37 | R=-0.043, p=0.89 |
|  | Right | Control | R=0.05, p=0.87 | R=0.06, p=0.85 | R=-0.04, p=0.9 | R=-0.007, p=0.98 |
|  |  | Umami | R=-0.32, p=0.31 | R=-0.04, p=0.9 | R=-0.51, p=0.09 | R=-0.12, p=0.71 |
|  |  | Bitter | R=-0.46, p=0.13 | R=-0.28, p=0.37 | R=-0.06, p=0.85 | R=-0.009, p=0.98 |
|  |  |  |  |  |  |  |
